# Correlation of Pulse Wave Transit Time with Pulmonary Artery Pressure in a Porcine Model of Pulmonary Hypertension

**DOI:** 10.3390/biomedicines9091212

**Published:** 2021-09-13

**Authors:** Fabian Mueller-Graf, Jonas Merz, Tim Bandorf, Chiara Felicitas Albus, Maike Henkel, Lisa Krukewitt, Volker Kühn, Susanne Reuter, Brigitte Vollmar, Sven Pulletz, Stephan H. Böhm, Daniel A. Reuter, Amelie Zitzmann

**Affiliations:** 1 Department of Anaesthesiology and Intensive Care Medicine, University Medical Centre Rostock, Schillingallee 35, 18057 Rostock, Germany; jonas.merz@uni-rostock.de (J.M.); tim.bandorf@uni-rostock.de (T.B.); chiara.albus@uni-rostock.de (C.F.A.); maike.henkel@uni-rostock.de (M.H.); lisa.krukewitt@uni-rostock.de (L.K.); Sven.Pulletz@med.uni-rostock.de (S.P.); Stephan.Boehm@med.uni-rostock.de (S.H.B.); daniel.reuter@med.uni-rostock.de (D.A.R.); Amelie.Zitzmann@med.uni-rostock.de (A.Z.); 2 Rudolf-Zenker-Institute for Experimental Surgery, University Medical Center Rostock, 18057 Rostock, Germany; Susanne.Reuter@med.uni-rostock.de (S.R.); brigitte.vollmar@med.uni-rostock.de (B.V.); 3 Institute of Communications Engineering, University of Rostock, Richard-Wagner-Straße 31, 18119 Rostock, Germany; volker.kuehn@uni-rostock.de

**Keywords:** pulmonary artery pressure (PAP), pulmonary hypertension (PH), pulse wave transit time (PWTT), pulse arrival time (PAT), pulse wave velocity (PWV)

## Abstract

For the non-invasive assessment of pulmonary artery pressure (PAP), surrogates like pulse wave transit time (PWTT) have been proposed. The aim of this study was to invasively validate for which kind of PAP (systolic, mean, or diastolic) PWTT is the best surrogate parameter. To assess both PWTT and PAP in six healthy pigs, two pulmonary artery Mikro-Tip™ catheters were inserted into the pulmonary vasculature at a fixed distance: one in the pulmonary artery trunk, and a second one in a distal segment of the pulmonary artery. PAP was raised using the thromboxane A2 analogue U46619 (TXA) and by hypoxic vasoconstriction. There was a negative linear correlation between PWTT and systolic PAP (*r* = 0.742), mean PAP (*r* = 0.712) and diastolic PAP (*r* = 0.609) under TXA. During hypoxic vasoconstriction, the correlation coefficients for systolic, mean, and diastolic PAP were consistently higher than for TXA-induced pulmonary hypertension (*r* = 0.809, 0.778 and 0.734, respectively). Estimation of sPAP, mPAP, and dPAP using PWTT is feasible, nevertheless slightly better correlation coefficients were detected for sPAP compared to dPAP. In this study we establish the physiological basis for future methods to obtain PAP by non-invasively measured PWTT.

## 1. Introduction

In current guidelines, pulmonary hypertension (PH) is defined as a mean pulmonary artery pressure (mPAP) above 25 mmHg measured by right heart catheterisation at rest [1]. Untreated PH will lead to right heart, and finally to global heart failure, and is associated with a high mortality within one year [1]. However, treatment requires reliable measurement and monitoring of pulmonary artery pressure (PAP). To date, diagnosis of PH is based on right heart catheterisation, but this comprises high invasiveness. Unfortunately, current non-invasive methods like echocardiography or magnetic resonance imaging (MRI) are not accurate enough to confirm the diagnosis of PH or serve as follow-up [1,2,3]. Therefore, it would be desirable to develop novel techniques for diagnosis and longitudinal monitoring of PH.

To replace direct pressure measurements within the pulmonary artery, surrogates need to be employed.

For systemic blood pressure, the idea of using pulse arrival time (PAT), the time delay between the R-spike of the electrocardiogram (ECG) and the arrival of the pulse wave at the finger plethysmograph, already emerged in the 1970s [4,5]. Applying a similar approach, Proença et al. estimated systolic PAP (sPAP) by PAT, but instead of the finger plethysmograph they determined the arrival of the pressure pulse within the pulmonary artery tree by electrical impedance tomography (EIT). In healthy volunteers, they induced acute PH by hypoxic vasoconstriction and determined sPAP by echocardiography [6]. These echocardiography-derived sPAP values ranged from 24 to 39 mmHg, showing an inverse linear correlation with PATs of 64 to 34 ms, despite a large interindividual variability [6]. Therefore, in another setup of 30 healthy volunteers, EIT-derived sPAP values have been adjusted by an individual offset for each participant [7]. In 2017, the same group published a computer simulation of a thoracic 4D (3D + time) bio-impedance model of the thorax, heart, aorta, and lungs including pulmonary vasculature. The simulated EIT data revealed a non-linear correlation between increasing mPAP (from 15 to 64 mmHg) and decreasing pulse wave transit time (PWTT) (from 56 to 12 ms) [8]. 

**Figure 1:**
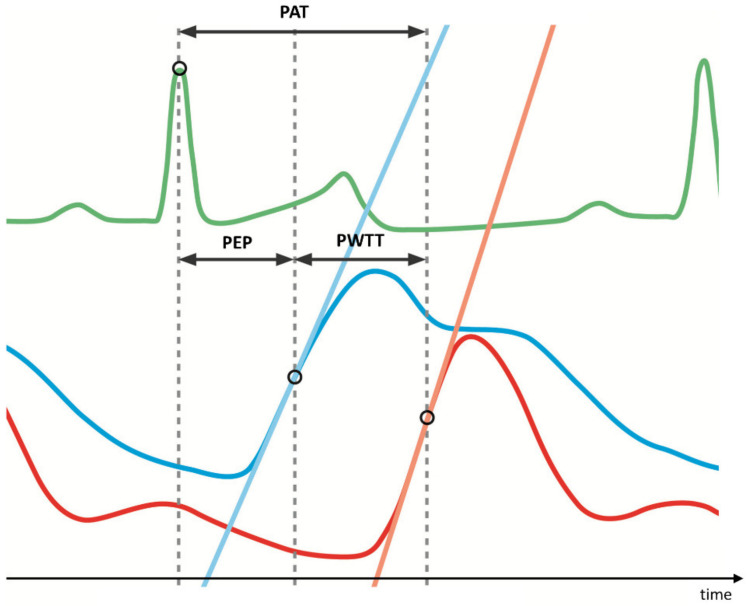
PAT is the sum of PEP (pre-ejection period—details see below) and PWTT. The pulse arrival time (PAT) is defined as the time interval between the R-spike of the ECG (green) and the pulse arrival at the distal pulmonary artery catheter (red). In this example the timespan between the R-spike of the ECG and the pulse arrival in the proximal pulmonary artery (blue) is equivalent to the PEP, as the catheter is placed right behind the pulmonary valve. The pulse propagation from the proximal pulmonary artery (blue) to the distal pulmonary artery (red) is called pulse wave transit time (PWTT). This PWTT, as well as the PAT, can be calculated without knowledge of the exact localization of the respective pressure probes. When the exact distance between the proximal and the distal catheter position ($\bar{\mathrm{PD}}$) is known, pulse wave velocity (PWV) can be calculated as: PWV = $\bar{\mathrm{PD}}$/PWTT [10].

Figure 1 provides an overview and explanations of the key parameters of interest for this article such as pulse wave transit times (PWTT), pre-ejection period (PEP), pulse arrival time (PAT), and pulse wave velocity (PWV) applicable for systemic and pulmonary circulation alike [9].

Summarising today’s literature, both linear [6,7] and non-linear [8] relationships between time-based surrogates and pulmonary artery pressures have been suggested. Furthermore, in systemic circulation, PWTT correlated either with systolic, mean, or diastolic pressures [10,11,12]. From these diverse findings, no specific inferences for the pulmonary circulation can be drawn.

Higher PWVs in the pulmonary artery have been associated with elevated pulmonary artery pressures due to mitral valve stenosis and pulmonary arterial stiffness [13,14,15]. Moreover, shorter PWTT is known to be a marker of pulmonary artery stiffness and is found in patients with idiopathic PH [16]. We therefore postulated that an inverse correlation between blood pressure and PWTT should also apply to the pulmonary vascular bed. However, to our knowledge, no study exists which validates acute PWTT changes over a wide range of pulmonary artery pressures within identical subjects.

Hence, the aim of this experimental study was to record PWTT directly by two pulmonary artery catheters in a porcine model of drug-induced acute PH to establish the physiological basis for developing new non-invasive methods for PAP estimation by PWTT.

## 2. Materials and Methods

### 2.1. Animal Model and Anaesthesia

The study was approved by the governmental ethical board for animal research (Landesamt für Landwirtschaft, Lebensmittelsicherheit und Fischerei, Mecklenburg-Vorpommern, Germany; No: 7221.3-1-037/19, 29 August 2019) and was carried out in accordance with the EU-directive 2010/63/EU and the Animal Research: Reporting of In Vivo Experiments guidelines 2.0 (ARRIVE 2.0) [17]. Six healthy German Landrace pigs (24.4–48.3 kg, 12–15 weeks old) were cared for and premedicated according to internal standards of the Institute for Experimental Surgery at Rostock University Medical Centre [18]. Animals were raised by local swine farmers and transferred to the housing facilities a week prior to the experiments for acclimatizing. They were given free access to standard laboratory chow and water. The initial state (animals anesthetized, prior to intervention) served as control; therefore, no randomisation was needed. The investigators were not blinded.

For premedication animals received an intramuscular injection of 8 mg/kg body weight azaperone (Stresnil™, Elanco, Cuxhaven, Germany) and 20 mg/kg body weight ketamine (10% Ketamin, Medistar Arzneimittelbetrieb GmbH, Ascheberg, Germany).

Upon arrival in the operating room, two peripheral venous catheters were placed in the veins of both ears. After preoxygenation, anaesthesia was induced using 0.2 mg fentanyl (Fentanyl-Janssen™, Janssen-Cilag, Neuss, Germany), 100 mg propofol (Propofol-Lipuro™, B. Braun, Melsungen, Germany) and 4 mg pancuronium (Inresa Arzneimittel GmbH, Freiburg, Germany) and maintained by continuous intravenous infusion of 4–8 mg kg^−1^ h^−1^ propofol, 5–10 µg kg^−1^ h^−1^ fentanyl, 6.4 mg h^−1^ pancuronium and 0.1 mg kg^−1^ h^−1^ midazolam (Dormicum™, Hoffmann La Roche AG, Grenzach-Wyhlen, Germany). The pigs were intubated endotracheally (ID 7.0 mm) and mechanically ventilated in a pressure-controlled mode using a Dräger Primus ventilator (Dräger Medical, Lübeck, Germany) with tidal volumes of 6 mL/kg and a PEEP of 5 mbar. Respiratory rate was adjusted to maintain end-tidal partial pressure of CO_2_ at 5 ± 0.4 kPa. Animals were euthanized using 45 mg/kg body weight pentobarbital (Release™, Wirtschaftsgenossenschaft deutscher Tierärzte eG, Garbsen, Germany) after completing all experimental steps.

### 2.2. Instrumentation

**Figure 2:**
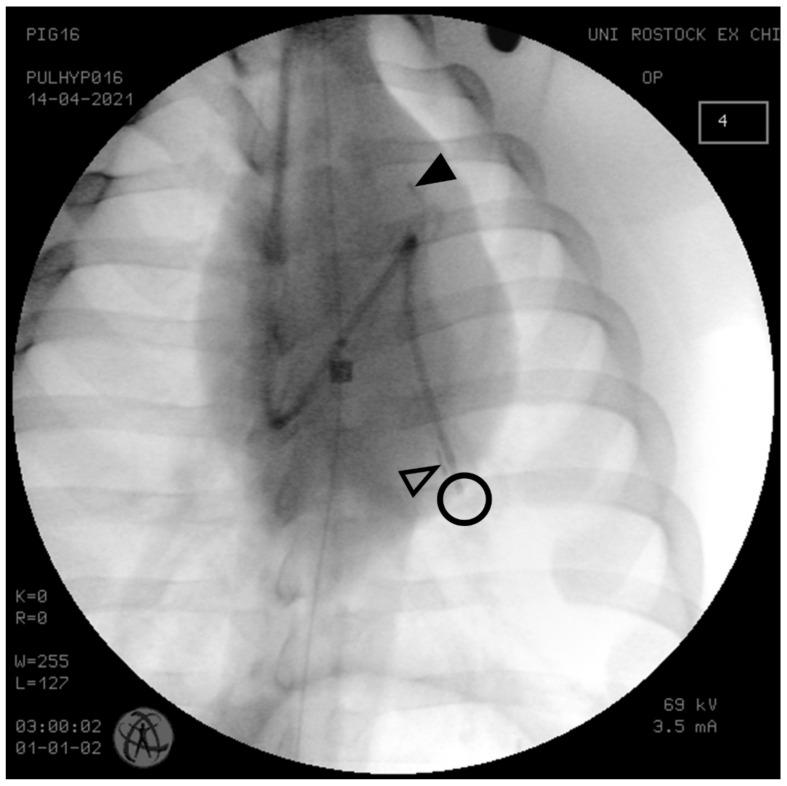
X-ray of the thorax to assess correct positions of catheters. A conventional pulmonary artery (Swan-Ganz) catheter was placed in the pulmonary artery for gold standard measurement of pulmonary artery pressure (circle). The sensors of two Millar Mikro-Tip catheters are located in a proximal (filled triangles) and a distal branch of the (open triangles) pulmonary artery (, X-ray taken with the C-Arm Ziehm Vision, Ziehm Imaging, Nuremberg, Germany).

For PAP measurement, a 7.5 Fr thermodilution pulmonary artery catheter (Arrow, Teleflex, Wane, PA, USA) was inserted into the proximal left pulmonary artery through an 8.5 Fr vascular sheath (Arrow, Teleflex, Wane, PA, USA) placed in the right external jugular vein. Two 5 Fr Mikro-Tip™ pressure catheters (Millar SPR-350, Millar Instruments Inc., Houston, TX, USA) were surgically placed in the right internal jugular vein and advanced into the left pulmonary artery, one immediately after the pulmonary valve (proximal), the other one in a peripheral branch of the pulmonary artery (distal) (Figure 2). The correct positions of intravascular catheters were verified by posterior-anterior X-ray taken with the C-Arm Ziehm Vision (Ziehm Imaging, Nuremberg, Germany).

### 2.3. Induction of PH

The thromboxane A2 (TXA) analogue U46619 (Enzo Life Sciences Science GmbH, Lörrach, Germany) was administered at doses of up to 0.15 µg kg^−1^ min^−1^ to induce PH by pulmonary vasoconstriction [19]. Temporary hypoxic vasoconstriction was induced by reducing FiO2 between 0.15 and 0.10 using Nitrogen (unavailable in one animal, ALPHAGAZ™, Air Liquide Deutschland, Düsseldorf, Germany) [20]. 

### 2.4. Data Acquisition and Processing

Data was acquired at 10 kHz using bridge transducer amplifiers in combination with dedicated hard- and software (PowerLab 16/35, and LabChart 8, both ADInstruments, Dunedin, New Zealand). Data with obvious external mechanical noise, transducer bouncing to pulmonary artery or pulmonary valve were not analysed. Premature heartbeats were excluded from further analyses.

**Figure 3:**
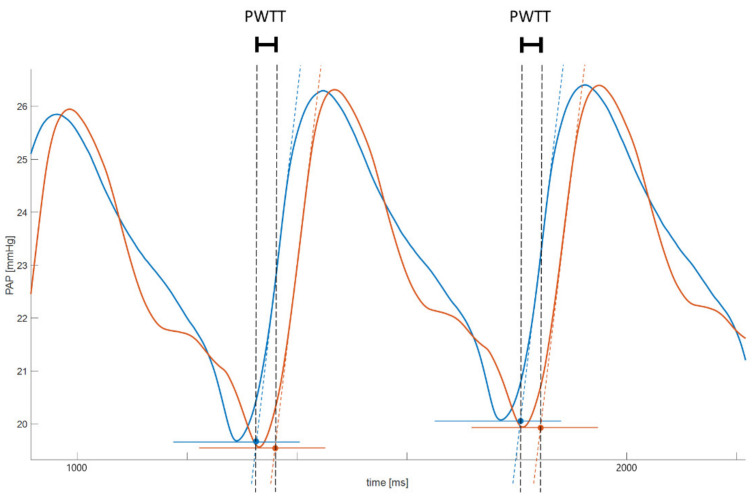
The pulse wave transit time between the proximal and distal pulmonary artery. The proximal pressure curve of the pulmonary artery is shown in blue, the distal one in red. Solid lines represent the pulmonary artery pressure in mmHg. Local pulse arrival was determined by intersection of the tangent of the local maximum derivative (dotted lines in blue and red) and the previous dPAP. Pulse wave transit time (PWTT) was calculated as the time difference between selected pulse arrival from the proximal and distal pressure curve (black dotted lines).

Stored data was exported from LabChart into a Matlab-compatible format (MATLAB^TM^ R2019b, The MathWorks, Inc., Natick, MA, USA). The local pulse arrival time was defined by the moment yielded by the intersection of a line tangent to the maximum systolic upstroke and a horizontal line through the previous diastolic PAP [21,22]. Proximal and distal pressure curves were analysed equally and separately. Pressure curves were filtered using a standard moving average filter over 1.000 datapoints. The first derivative of this fitted pressure curve was calculated and filtered using another moving average filter over 100 datapoints. A standard Matlab routine for local maxima detection was used to determine the time point of maximum inclination of each heartbeat-related PAP oscillation (Figure 3).

PWTT was then calculated as the time difference between the arrival of the pressure wave at the distal and the proximal site. Thereafter, PWTT was correlated with systolic, mean and diastolic PAP derived from pulmonary artery catheter.

The distance between the sensors of both Mikro-Tip pressure catheters was approximated by measuring the outer distance of the plug of the pressure transducers as reported previously [15]. X-ray was used to exclude loops in the path of both Millar Mikro-Tip catheters. PWV was calculated by dividing this distance by the PWTT. The data presented and the Matlab code used in this study are available on request from the corresponding author. The data are not publicly available due to copyright issues.

### 2.5. Statistics

Linear regression analysis was performed with the least square method and the respective correlation coefficient was calculated using SigmaPlot 12.0 (Systat Software, Inc., San Jose, CA, USA). For each linear regression, F-Test was performed and a p-value calculated. After Fisher’s-Z transformation weighted mean correlation in each group was calculated and retransformed in r coefficient [23]. Standard Error for correlation coefficient was calculated as previously recommended [23]. Repeated measurement ANOVA (rmANOVA) was used for detection of statistically significant differences in the calculated correlation coefficients (*p* ≤ 0.05).

## 3. Results

Baseline sPAP, mPAP and dPAP were 28.7 ± 7.3, 22.4 ± 6.0 and 15.0 ± 4.9 mmHg before TXA- and 23.7 ± 2.7, 18.0 ± 2.1 and 11.6 ± 1.2 mmHg before hypoxia-induced PH (mean ± SD). For each animal the individual baseline is given in Table 1 together with the respective PWTT and the PWV under resting conditions. After PH induction with TXA sPAP, mPAP and dPAP were 43.7 ± 10.7, 32.9 ± 7.1, and 21.8 ± 4.3 mmHg (mean ± SD); during hypoxia-induced PH values were 38.5 ± 5.7, 29.9 ± 4.3, and 22.1 ± 3.5 mmHg (mean ± SD). Individual values for each animal are given in Table 2.

**Figure 4:**
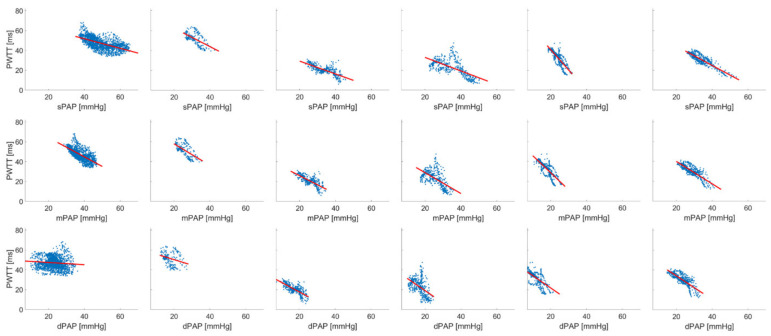
Inverse linear correlation between sPAP, mPAP, dPAP and PWTT during thromboxane A2-induced PH in six pigs. Average correlation coefficient was calculated via Fisher’s-Z transformation (sPAP *r* = 0.742 ± 0.104, mPAP *r* = 0.712 ± 0.078 and dPAP *r* = 0.609 ± 0.179). Linear regression was performed by least square method.

**Figure 5:**
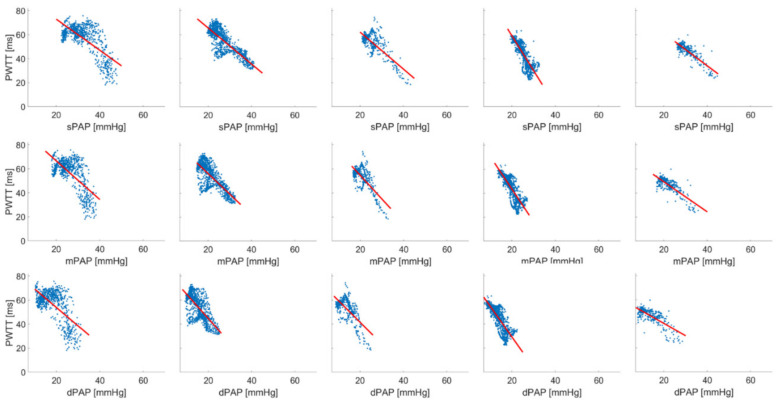
Inverse linear correlation between sPAP, mPAP, dPAP and PWTT during hypoxic pulmonary vasoconstriction in five pigs. Average correlation coefficient was calculated via Fisher’s-Z transformation (sPAP r = 0.809 ± 0.081 *, mPAP r = 0.778 ± 0.082 and dPAP r = 0.734 ± 0.090, *: Significant difference between sPAP vs. dPAP, *p* ≤ 0.05, rmANOVA). Linear regression was performed by least square method.

Figure 4 and Figure 5 show the inverse linear correlation between PWTT and PAP induced by U46619 and under hypoxia. A mean correlation coefficient was calculated with standard error under TXA for sPAP *r* = 0.742 ± 0.104, mPAP *r* = 0.712 ± 0.078 and dPAP *r* = 0.609 ± 0.179 (Table 3, A, *n* = 6) as well as for hypoxic condition with sPAP *r* = 0.809 ± 0.081, mPAP *r* = 0.778 ± 0.082 and dPAP *r* = 0.734 ± 0.090 (Table 3, B *n* = 5). Using a rmANOVA a significantly better correlation for sPAP compared to dPAP in the hypoxia treatment group was found.

## 4. Discussion

It was our aim to characterise the physiology of the PWTT with regards to sPAP, mPAP and dPAP. During induction of PH and mechanical ventilation, PWTT showed a good linear correlation with sPAP, mPAP and dPAP, which was slightly better during hypoxia. Due to the large sample size of each measurement period the correlation coefficient passed the F-Test and presented with *p* ≤ 0.001. Although the literature provides inconsistent information on the relationship between PWTT and systolic, mean, or diastolic pressures within both, pulmonary and systemic circulation, most publications report their strongest correlations for systolic and mean pressure [10,11,12,15,24,25,26,27,28]. These findings are in accordance with our results in pulmonary circulation, which also showed better correlations for sPAP compared to dPAP in the hypoxia group. Under induction of PH with TXA, no significant differences between the correlation coefficients could be derived. Nevertheless, corelations between sPAP, as well as mPAP and PWTT were strong, and moderate for dPAP for TXA.

In their 2016 study, Proença et al. reported a linear correlation of PAT with sPAP during acute hypoxia [6]. In 2017, however, a non-linear correlation was reported for their simulation study, where PWTT and mPAP were modelled even at mPAP values as high as 65 mmHg [29]. Both findings were explained by the alterations of vascular compliance in patients with PH. In his most recent paper Proença assumes a linear relationship of PAT and sPAP for mildly to moderately increased sPAP values referring to the elastic components of the pulmonary vasculature and suggesting a non-linear behaviour for higher sPAP values [7]. Intravascular ultrasound studies in humans showed an abnormal stiffness of the pulmonary artery for patients with mPAP values above 35–40 mmHg, when presumably all elastic fibres are fully stretched [30]. This could lead to non-linear behaviour for mPAP values over 40 mmHg. Nevertheless, these findings have to be interpreted cautiously. Due to the nature of chronic PH, it remains unclear which part of the decreased compliance is due to vascular remodelling and which by fully distending the elastic fibres within the pulmonary artery vessel wall. Based on the characteristic shape of their compliance curves Lau et al. could distinguish two groups of patients: those with and without chronic PH (compare Figure 4 in Lau et al., 2012) [30].

Referring to the abovementioned literature, we analysed our data for linear and non-linear behaviour, using linear fitting by Pearson’s least square method and the polynomial fit function of Matlab with 2 degrees of freedom as described by Lau [30]. Linear correlations were the ones that described the relationship between PWTT and PAP for both, TXA- as well as for hypoxia-induced PH the best. Reproducible or conclusive non-linear behaviours were not identified. Therefore, we assume the relationship between PWTT and mPAP to be linear for the tested pulmonary pressures below 40 mmHg. Higher mPAP values could not be investigated in our experimental model as higher doses of TXA caused right heart failure. Our finding of a linear relationship corresponds with the recent literature which describes a transition of compliance from linear to non-linear behaviour at mPAP levels higher than 40 mmHg [30]. In a pilot study, we could demonstrate a linear correlation of PWTT and mPAP after subtracting the highly variable PEP [6,7,29,30,31].

The subset of experiments during hypoxia was conducted for two reasons: (1) knowing that hypoxia increases PAP due to hypoxic pulmonary vasoconstriction; (2) to exclude possible variations in PWTT by TXA itself. The slightly higher correlation between mPAP and PWTT during hypoxia can be explained by the more stable heart rate under short hypoxia and lack of the TXA-related chronotropic effects [32]. Nevertheless, using the TXA analogue U46619 is highly recommended for reversible PH induction in porcine models [32]. During hypoxic pulmonary vasoconstriction, we obtained a smaller range of pulmonary hypertension, knowing that higher variance of PWTT occurs especially under very high pressures (Figure 5).

Kopeć et al. assessed PWV in 26 patients with PH and 10 healthy control patients using the foot-to-foot velocity method in sequential recordings of the pulmonary artery pulse waveform at two different sites in the pulmonary artery tree and found higher PWV in patients with PH [15]. It remained unclear whether the higher PWV was induced by higher pulmonary artery pressures or caused by stiffer pulmonary vessels due to pulmonary artery remodelling [15,33]. Considering the results of our model of acute PH, we assume that one part of this increase of PWV seen by Kopeć et al. was caused by a faster pulse propagation within the pulmonary artery caused by higher pressures. Furthermore, the range of PWV from 3.5 ms^−1^ to 10 ms^−1^ reported by Kopeć corresponds well with ours [15].

Even though our study presents a strong correlation of PWTT with sPAP, mPAP and dPAP as measured by the gold standard of pulmonary artery catheterisation, this experiment has clear limitations. The definition of pulse wave arrival is complex. Pressure curve shapes change differently between proximal and distal pressure curves under pulmonary hypertension. This could lead to false shortening of PWTT. We decided for an automated calculation, using an intersecting tangent method because of its relative robustness compared to maximal systolic upstroke and local minimum techniques [22]. Furthermore, the fully automated analysing algorithm excludes observer influences. Exact intravital distance of both pressure transducers, and thereby PWV, could neither be derived by distance of outer plugs nor by X-ray alone [15]. With a combination of both techniques a possible major error could be excluded. Nevertheless, our values for PWV are within the range reported in previous studies [15,34].

Nowadays, according to the 2015 ESC/ERS guidelines, the diagnostic process for PH starts with the suspicion of PH based on medical history, symptoms, and echocardiography results compatible with PH [1]. If PH is considered at least likely, this triggers a series of invasive diagnostics including right heart catherization (RHC). A 2018 update on definition, clinical classification and initial diagnosis of PH by the German Society of Cardiology, the German Respiratory Society and the German Society of Pediatric Cardiology mentions less invasive modalities such as cardiac magnetic resonance imaging (CMRI), as newer studies show good correlations of MRI-derived hemodynamic parameters with invasively measured values in RHC [34]. This comprises estimation of mPAP using ventricular mass index and interventricular septal angle, including RV function along with right atrial size, but these indirect estimates based on multiple variables always bear possible sources of error [35]. A more direct method for estimating mPAP by CMRI is assessing the presence and duration of vortical blood flow, i.e., a concentric ring shaped flow, in the main pulmonary artery [36].

Nevertheless, MRI-derived findings for PWV are not conclusive. Laffon proposed a MR-based phase mapping in the main pulmonary artery for PWV calculation [37]. PWV correlated in his cohort of 15 patients with mPAP (correlation coefficient *r* = 0.68). Comparing various CMRI based techniques for mPAP calculation Roeleveld could not confirm these findings [38]. All CMRI techniques involve an ECG-gating to extrapolate one representative cardiac cycle from around 20–40 image frames. The resulting temporal resolution is approximately 25 to 50 ms. In our study the PWTT at baseline was around 50 ms and was reduced by around 20% to 40%; thus, 10 to 20 ms during PH renders such MRI-based time surrogates inappropriate due to the low sampling rate. Until the time when MRI scans can be taken at rates higher than 200 Hz, other indirect surrogates, such as the quantification of pressure-induced ventricular deformations described by Swift, appear to be more suitable for estimating pulmonary artery pressures.

## 5. Conclusions

Using the gold standard of two independent invasive catheters we found a strong negative linear correlation between the PWTT and sPAP, mPAP and dPAP over a wide range of pressures in a porcine model of acute PH. Correlation of PWTT with dPAP was slightly lower than with sPAP. In this study we established the physiological basis for developing future methods based on PWTT to obtain reliable repeated PAP values by non-invasive means.

## Figures and Tables

**Figure 6:**
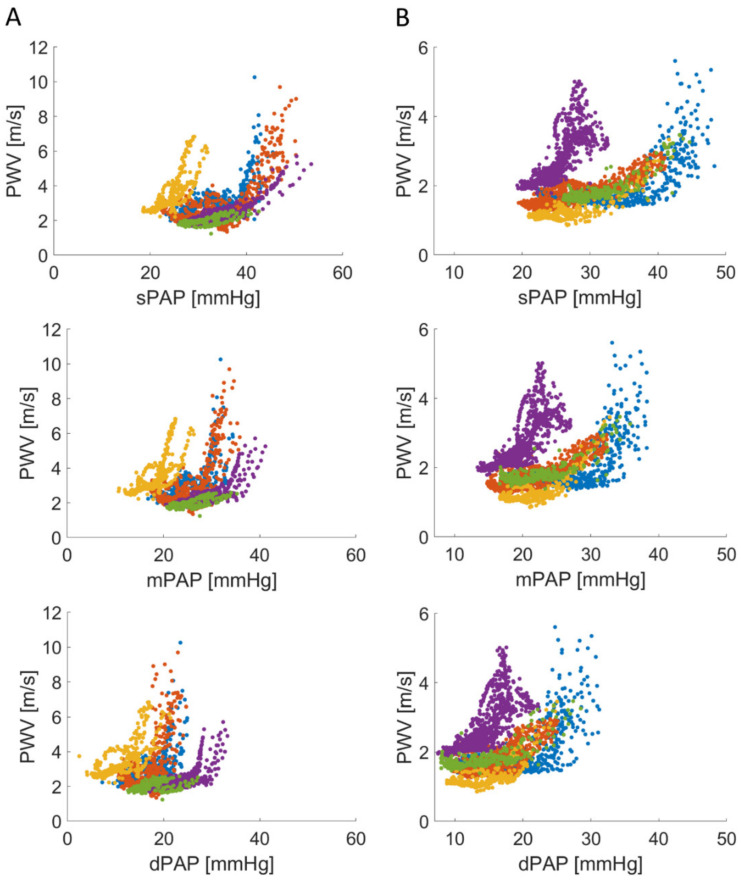
Hyperbolic correlation between sPAP, mPAP, dPAP and PWV during thromboxane A2-induced PH ((**A**), *n* = 5, catheter distance unavailable in one animal) and hypoxic pulmonary vasoconstriction ((**B**), *n* = 5). PWV was calculated as pressure sensor distance divided by PWTT given in m/s. For each animal an individual colour was used.

**Table 1:** Baseline mean values with respective standard deviations for each animal at resting conditions before TXA- (A) and hypoxia-induced (B) PH. Missing data in animal 1 (n/a) are due to unavailable nitrogen gas and missing catheter distance in this experiment.

**A**	**sPAP [mmHg]**	**mPAP [mmHg]**	**dPAP [mmHg]**	**PWTT [ms]**	**PWV [m/s]**
1	42.5 ± 2.9	33.0 ± 1.9	22.5 ± 2.8	54.9 ± 3.4	n/a
2	28.7 ± 2.3	23.2 ± 2.0	15.9 ± 2.5	55.1 ± 4.1	1.9 ± 0.1
3	25.6 ± 1.3	20.1 ± 1.5	11.9 ± 1.4	27.2 ± 2.2	2.3 ± 0.2
4	25.0 ± 2.2	19.5 ± 1.8	12.6 ± 1.5	24.2 ± 2.4	2.7 ± 0.3
5	21.5 ± 1.6	15.0 ± 2.1	9.3 ± 2.7	38.6 ± 2.7	2.7 ± 0.2
6	29.1 ± 1.8	23.7 ± 2.0	18.6 ± 2.5	35.5 ± 2.9	2.0 ± 0.2
**B**	**sPAP [mmHg]**	**mPAP [mmHg]**	**dPAP [mmHg]**	**PWTT [ms]**	**PWV [m/s]**
1	n/a	n/a	n/a	n/a	n/a
2	25.0 ± 2.0	20.5 ± 1.9	13.5 ± 2.5	62.6 ± 4.8	1.6 ± 0.1
3	21.8 ± 1.8	16.4 ± 1.6	12.0 ± 2.0	57.8 ± 7.2	1.6 ± 0.2
4	22.9 ± 1.6	18.4 ± 1.7	11.6 ± 2.5	54.2 ± 5.3	1.2 ± 0.1
5	21.1 ± 1.2	15.4 ± 1.6	10.0 ± b1.8	55.6 ± 2.3	2.0 ± 0.1
6	27.9 ± 1.7	19.5 ± 2.2	11.2 ± 3.1	47.9 ± 3.4	1.7 ± 0.1

**Table 2:** Mean values with respective standard deviations for each animal after PH induction either by TXA (A) or by hypoxia (B). Missing data in animal 1 (n/a) are due to unavailable nitrogen gas and missing catheter distance in this experiment.

**A**	**sPAP [mmHg]**	**mPAP [mmHg]**	**dPAP [mmHg]**	**PWTT [ms]**	**PWV [m/s]**
1	60.4 ± 3.1	43.9 ± 1.8	20.0 ± 5.6	42.9 ± 3.3	n/a
2	37.5 ± 2.2	30.8 ± 2.1	21.6 ± 2.9	42.6 ± 0.7	2.4 ± 0.2
3	42.1 ± 1.9	31.4 ± 1.6	22.3 ± 1.5	13.4 ± 4.1	5.0 ± 1.5
4	47.3 ± 1.4	32.4 ± 1.6	21.3 ± 1.7	10.2 ± 2.0	6.5 ± 1.2
5	28.4 ± 1.8	22.3 ± 2.1	16.3 ± 2.8	21.0 ± 5.2	5.3 ± 1.2
6	46.8 ± 4.4	36.6 ± 2.7	29.5 ± 2.3	16.0 ± 3.5	4.5 ± 0.8
**B**	**sPAP [mmHg]**	**mPAP [mmHg]**	**dPAP [mmHg]**	**PWTT [ms]**	**PW [m/s]**
1	n/a	n/a	n/a	n/a	n/a
2	44.8 ± 1.5	35.1 ± 1.7	27.1 ± 2.0	30.8 ± 6.0	3.4 ± 0.7
3	38.4 ± 1.4	29.6 ± 1.5	20.6 ± 2.1	38.0 ± 3.8	2.5 ± 0.2
4	39.7 ± 2.1	29.5 ± 2.4	21.0 ± 3.2	26.5 ± 6.7	2.6 ± 0.6
5	29.2 ± 1.8	23.5 ± 1.8	17.9 ± 1.9	33.0 ± 4.2	3.5 ± 0.5
6	40.5 ± 3.4	31.8 ± 2.9	23.7 ± 2.9	30.2 ± 6.3	2.8 ± 0.5

**Table 3:** Statistics for linear regression analysis for correlation of PWTT and sPAP, mPAP and dPAP for thromboxane A2-induced PH (A, n = 6) and hypoxic pulmonary vasoconstriction (B, n = 5). For each animal the number of consecu-tive heart beats analysed during induction of PH is given as n. Linear regression was performed using the least square method by Pearson, F-Test and *p* value were calculated (below). The significantly better correlation for sPAP compared to dPAP is highlighted with the asterisk (*) (*p* ≤ 0.05, rmANOVA).

**A**		**sPAP**				**mPAP**				**dPAP**			
**Pig**	**n**	**Linear** **Regression**	**r^2^**	**F**	***p***	**Linear** **Regression**	**r^2^**	**F**	***p***	**Linear** **Regression**	**r^2^**	**F**	***p***
1	1611	PWTT = 70.945 − (0.481 * sPAP)	0.356	888.151	<0.001	PWTT = 83.645 − (0.969 * mPAP)	0.499	1601.15	<0.001	PWTT = 49.662 − (0.112 * dPAP)	0.00885	14.364	<0.001
2	686	PWTT = 98.938 − (1.298 * sPAP)	0.502	689.041	<0.001	PWTT = 99.054 − (1.616 * mPAP)	0.466	596.726	<0.001	PWTT = 84.597 − (1.537 * dPAP)	0.430	517.007	<0.001
3	333	PWTT = 42.133 − (0.646 * sPAP)	0.547	400.226	<0.001	PWTT = 44.194 − (0.917 * mPAP)	0.534	379.604	<0.001	PWTT = 37.053 − (0.967 * dPAP)	0.542	391.071	<0.001
4	377	PWTT = 46.632 − (0.683 * sPAP)	0.399	248.757	<0.001	PWTT = 44.595 − (0.815 * mPAP)	0.210	90.716	<0.001	PWTT = 43.850 − (1.239 * dPAP)	0.245	121.935	<0.001
5	426	PWTT = 81.659 − (2.047 * sPAP)	0.649	783.579	<0.001	PWTT = 62.936 − (1.719 * mPAP)	0.597	628.24	<0.001	PWTT = 46.610 − (1.243 * dPAP)	0.447	342.462	<0.001
6	440	PWTT = 63.723 − (0.972 * sPAP)	0.765	1422.27	<0.001	PWTT = 62.645 − (1.129 * mPAP)	0.686	955.157	<0.001	PWTT = 58.002 − (1.188 * dPAP)	0.628	738.206	<0.001
**B**		sPAP *				**mPAP**				**dPAP**			
**Pig**	**n**	**Linear** **Regression**	**r^2^**	**F**	***p***	**Linear** **Regression**	**r^2^**	**F**	***p***	**Linear** **Regression**	**r^2^**	**F**	***p***
1	686	PWTT = 98.938 − (1.298 * sPAP)	0.502	689.041	<0.001	PWTT = 99.054 − (1.616 * mPAP)	0.466	596.726	<0.001	PWTT = 84.597 − (1.537 * dPAP)	0.430	517.007	<0.001
2	1022	PWTT = 95.676 − (1.501 * sPAP)	0.671	2083.98	<0.001	PWTT = 90.743 − (1.719 * mPAP)	0.598	1518.03	<0.001	PWTT = 85.160 − (2.027 * dPAP)	0.503	1030.82	<0.001
3	444	PWTT = 93.021 − (1.542 * sPAP)	0.597	653.743	<0.001	PWTT = 93.907 − (1.967 * mPAP)	0.547	534.506	<0.001	PWTT = 77.779 − (1.806 * dPAP)	0.467	386.649	<0.001
4	1040	PWTT = 117.075 − (2.902 * sPAP)	0.750	3114.64	<0.001	PWTT = 97.682 − (2.717 * mPAP)	0.730	2804.00	<0.001	PWTT = 80.169 − (2.548 * dPAP)	0.709	2531.34	<0.001
5	290	PWTT = 88.233 − (1.354 * sPAP)	0.716	725.429	<0.001	PWTT = 74.310 − (1.249 * mPAP)	0.652	539.333	<0.001	PWTT = 60.839 − (1.024 * dPAP)	0.551	354.094	<0.001

PWV ranged between 1.24 ms^−1^ and 10.25 ms^−1^ for TXA-induced pulmonary hypertension and between 0.86 ms^−1^ and 5.61 ms^−1^ for hypoxic conditions (Figure 6). Each animal showed a hyperbolic configuration of PWV plotted against PAP.

## Data Availability

The data presented and Matlab code in this study are available on request from the corresponding author. The data are not publicly available due to copyright issues.
